# Supplementary material for: Drivers of land-use changes in societies with decreasing populations: A comparison of the factors affecting farmland abandonment in a food production area in Japan
Source: PLoS One. 2020 Jul 24;15(7):e0235846. doi: 10.1371/journal.pone.0235846 (PMC7380605; doi:10.1371/journal.pone.0235846)
Supplement: Attachment — Submitted filename: ResponseToReviewer_PLOSONE_20200505.doc [file pone.0235846.s003.doc]

Dear Reviewers

Thank you for constructive comments on our manuscript. We have revised the manuscript accordingly.

**Reviewer #1**

1. Comment 1: Manuscript is well written and clearly describes the factors of land use changes in Japanese Society by comparing the factors affecting the farmland abandonment. Anyhow add more citations in support of your results in discussion section.

We referenced additional studies to support our results in the discussion section. In particular, we have cited studies showing the important determinants of farmland abandonment in the population decline phase. These determinants include disadvantaged geographical conditions, depopulation, and low accessibility to markets.

**Reviewer #2**

2. Comment 2: The author need to provide a section on more recent literature

We added recent literature in the Introduction, Discussion and Concluding remarks; thus, we revised the description of the world’s population dynamics (L30 – L33) and removed Fig. 1 (Total population of the world, 1950-2010 (Estimates) and 2011-2100 (Projections) provided by the United Nations (2014)) because a similar figure is found in the latest report of world population prospects provided by the United Nations (2019) (Reference 3).

3. Comment 3: In the discussion section, no comparison with the literature was developed

We have referenced several studies and compared our findings with the results of previous studies in the Discussion (see the response to Comment 9 for details.).

4. Comment 4: The paper needs to elaborate more the contribution in terms of methodology and added value to the literature

We have mentioned the potential and value of our results in the Abstract (L21 – L22) and Discussion (L434 – L437). Our results showing the land-use drivers associated with specific periods may provide new insights for other developed countries experiencing depopulation problems. We have also described the political implications of our results in the Concluding remarks (see the response to Comment 5 for details.). Since this is not a methodology paper, we did not discuss the methods.

**Reviewer #3**

5. Comment 5: the authors indicate that the study should be helpful to facilitate future land planning, but they say little about recommendations or policy implications of their findings and discussion. Do they have an overall assessment of the strengths and weaknesses of the polices through the various periods?

We have discussed the political implications of our results (L393 – L401; L424 – L433). Our results showed that the disadvantaged farmlands, such as marginal, underproductive, narrow, and steep lands, will be continuously abandoned even in the decline phase. These geographical conditions were similar to eligible farmlands for the direct payment (DP) policy of subsidies to maintain unfavorable farmlands in place since 2000 in Japan. Thus, the current DP system might be effective even during the population decline phase. However, the population decline has become one of the determinants for farmland abandonment in recent years, and therefore, we expect that abandoned farmlands will increase in the future with depopulation. According to a previous study, population decentralization mitigates farmland abandonment even in the depopulation scenario; therefore, abandoned farmlands might be reduced using such a land management policy. The land management policy for population decentralization that was initiated in 2016 in Hokkaido would be a key policy for mitigating farmland abandonment.

6. Comment 6: as for ecological implications of farmland abandonment (and potential for recovery of ecosystems), this topic is mentioned a few times but there is no clear assessment or recommendations around this. The conclusion mentions two possible outcomes of reverting to natural ecosystems or becoming a wasteland. Could the authors comment on how common each of these is for the lands that have been abandoned to date?

We have discussed the ecological implications in the Concluding remarks (L448 – L461). The previous studies showed that farmland abandonment can have either negative or positive ecological effects depending on the conservation target and regional circumstances. We have discussed previous studies to show that natural succession in abandoned farmland is considered an opportunity or a threat to regional biodiversity.

7. Comment 7: Reference 13 relates to "Rewilding abandoned landscapes in Europe". Has this been explicitly attempted in Japan?

We have described attempts to rewild abandoned farmlands in Japan in the Concluding remarks (L462 – L475). In Japan, there have been very few attempts to rewild abandoned farmlands because vegetation recovery in abandoned farmlands is considered to be a threat to agrobiodiversity represented by traditional agricultural activities. However, there are several activities to restore abandoned farmlands to original natural ecosystems in areas with high potential and needs for rewilding.

8. Comment 8: clarify why the analysis only extends to 2009

We have added an explanation for why we limited the latest period to include only data available up to 2009 in the Methods (L146 – L152). In Japan, the grid square system was used as a spatial unit for statistical use and/or biodiversity surveys beginning in 1969. However, there are two geographical summary grids based on old and new Japanese geodetic reference systems. Although the Japanese geodetic reference system was changed to the new one in 2002, the land-use data and population data had been continuously released using the two grids. However, the production of population data using old grid system was ended in 2010, and only data from the new grid system has been released since then. Therefore, we have to use the land-use datasets only up to 2009 to analyze the relationship between population and land-use using the same (old) grid system.

9. Comment 9: how common is farmland abandonment in other parts of the world? What are the similarities and differences compared to the Japanese case/context?

We referenced studies regarding farmland abandonment in the Discussion (L391 – L392). Our results showed that geographical conditions, such as marginal, underproductive, narrow, and steep slopes, were determinants of an increase in abandoned farmlands during the growth and the decline phases, which was a similar trend found by the previous studies in other countries conducted in the growth phase. An original finding of the present study is that social conditions, such as the distance from densely inhabited districts (DIDs) and the population, exerted opposite effects between the population growth and decline phases.
